# Physiological Responses to Basic Tastes for Sensory Evaluation of Chocolate Using Biometric Techniques

**DOI:** 10.3390/foods8070243

**Published:** 2019-07-05

**Authors:** Thejani M. Gunaratne, Sigfredo Fuentes, Nadeesha M. Gunaratne, Damir D. Torrico, Claudia Gonzalez Viejo, Frank R. Dunshea

**Affiliations:** 1 University of Melbourne, School of Agriculture and Food, Faculty of Veterinary and Agricultural Sciences, VIC 3010, Australia; 2 Lincoln University, Department of Wine, Food and Molecular Biosciences, Faculty of Agriculture and Life Sciences, Lincoln 7647, New Zealand

**Keywords:** basic tastes, sensory analysis, biometrics, emotions

## Abstract

Facial expressions are in reaction to basic tastes by the response to receptor stimulation. The objective of this study was to assess the autonomic nervous system responses to basic tastes in chocolates and to identify relationships between conscious and unconscious responses from participants. Panelists (*n* = 45) tasted five chocolates with either salt, citric acid, sugar, or monosodium glutamate, which generated four distinctive basic tastes plus bitter, using dark chocolate. An integrated camera system, coupled with the Bio-Sensory application, was used to capture infrared thermal images, videos, and sensory responses. Outputs were used to assess skin temperature (ST), facial expressions, and heart rate (HR) as physiological responses. Sensory responses and emotions elicited during the chocolate tasting were evaluated using the application. Results showed that the most liked was sweet chocolate (9.01), while the least liked was salty chocolate (3.61). There were significant differences for overall liking (*p* < 0.05) but none for HR (*p* = 0.75) and ST (*p* = 0.27). Sweet chocolate was inversely associated with angry, and salty chocolate positively associated with sad. Positive emotion-terms were associated with sweet samples and liking in self-reported responses. Findings of this study may be used to assess novel tastes of chocolate in the industry based on conscious and emotional responses more objectively.

## 1. Introduction

Cocoa-based products, especially chocolate, have become exceptional food products in terms of nutrition as well as in indulgence in the day-to-day life of consumers [1]. Chocolate is considered as a semi-solid suspension of fine solid particles obtained from sugar and cocoa in a continuous fat phase. Dark, milk, and white are the primary chocolate categories, which vary in cocoa butter, cocoa solids, and milk fat content. As a result of the variation in the proportions of the constituents, the final products differ in carbohydrate, fat, and protein content [2]. Hence, it is important for industries as well as for researchers to identify the sensory perceptions of chocolate, mainly the appearance, aroma, texture, and flavor [3].

Despite the extensive sensory and consumer tests being used, limitations of traditional sensory profiling and hedonic scales still lead to a high failure rate of new food products in the market [4]. Consumer choice is driven more by unconscious responses than conscious [5]. The total effect of messages to consumers (marketing, packaging, and sensory) is expressed back by 7% verbally, 38% vocally, and 55% through facial expressions (FE) and behavior [6] that may not be conscious responses. Unconscious responses may be measured by behavioral and physiological measurements, also known as biometrics, which are important in identifying the preference and purchase intention of consumers. Facial expressions (FE), skin temperature (ST), and heart rate (HR) are considered important parameters that could reflect emotions and liking towards food products and beverages [7].

A review conducted by Guillory et al. [8] stated that invasive biometric techniques express the emotional states using unique insights of neural activation. Furthermore, in a previous study, de Wijk et al. [9] used invasive techniques by attaching electrodes and sensors to the body of participants to measure HR, ST, and skin conductance. However, this method can be considered as disadvantageous due to the possible stress that participants feel before the tasting sessions. which can affect their conscious and unconscious responses by being constantly aware of the sensors [10,11]. Hence, determining HR using non-invasive video analysis can be conducted as an alternative and efficient method. HR assessment can be conducted using a photoplethysmography (PPG) technique, where videos of participants are captured to analyze changes in skin luminosity from the face by magnifying this signal through computer vision algorithms. This signal is related to the blood flow from and to the faces of participants [7,10]. For ST, thermal cameras are used to obtain the distribution of facial temperatures of panelists. The perception of different tastes can produce responses from the autonomic nervous system (ANS), causing changes in the blood flow and body temperature, which are used as indicators of the elicited emotions [12]. FE can be obtained through video analysis using computer vision algorithms to assess facial movements that are associated with different expressions of emotions. There is commercial software available that can provide this analysis from videos, such as Face Reader™ (Noldus Information Technology, Wageningen, Netherlands) [13]. Hence, in this study, biometrics such as HR, ST, and FE and hedonic measurements (overall liking, etc.) were obtained to determine the correlations between conscious and unconscious responses of panelists to chocolate.

Specific facial reactions are expressed during the exposure to basic tastes of foods when the sensory perceptions are registered via the activation of taste receptors in the mouth, tongue, or throat. Different foods contain tastants that give perceptions of one or combined basic tastes, including bitterness, saltiness, sourness, sweetness, or umami [14]. The sweet compounds are formed by simple carbohydrates, and associated facial reactions have been identified as pleasant expressions, such as relaxation, licking of lips, retraction of mouth angles, and lip sucking [15]. Sodium ions (Na^+^) are mainly responsible for providing saltiness, and a pure salt taste results from the interaction between sodium chloride (NaCl) and the taste receptors [14]. It has been shown that distinctive FE cannot be associated with saltiness. However, it can be linked to FE that are associated with negative feelings, such as pressing of lips, lengthening of the tongue, and gazing, which can be recorded [16]. Several chemical compounds are responsible for bitterness, and the responses from these compounds are related to evolutionary traits of humans to avoid poisonous foods [17]. The bitter taste elicits strong FE when compared to others, such as frowning, eye widening, brow lower, nose wrinkle, and upper lip raise [15]. Also, these reactions have been categorized as negative and unpleasant [14]. In general, acids are responsible for the sourness response of foods, which is caused when the taste receptors are activated by the hydrogen (H^+^) ions. FE related to sourness, including the closing of eyes, blinking, pressing of lips, and nose wrinkling, are considered negative [14]. The fifth basic taste used in this study was umami, and it is produced by monosodium glutamate (MSG), inosine monophosphate (IMP), or guanosine monophosphate (GMP), which are similar to tastes of savory foods, such as mushroom and meats [18]. Anions are responsible for the umami taste [19]; the detection of the carboxylate anion of glutamate is responsible for the sensation of umami [20]. FE associated with umami is considered neutral or pleasing [21]. Other recently acknowledged tastes, such as fat and starchy, were not considered in this study [22,23,24].

Previous studies have shown that different tastes of foods lead to variations in HR and ST that can be related to changes in the hedonic valence or emotions [25,26]. According to de Wijk et al. [27], increases in HR, ST, and skin conductance responses are generally associated with positive emotions, while negative emotions lead to increased HR and skin conductance and decreased ST.

The ANS responses are an essential aspect of emotional reactions according to many theories of emotions [28]. Moreover, several studies have shown the two-way effect between eating behavior and emotional responses; where food that people consume may affect their emotions, and human emotions may be responsible for the selection of foods that people tend to eat [29]. Hence, lexicons of emotions have been developed for food products to have an emotional metric system, which has played a vital role in food perception. For example, the EsSense Profile^®^ (Carmignano, Italy) was developed to quantitatively determine the intensities of each emotion using a 5-point hedonic scale (1 = not at all, to 5 = extremely) [30,31].

Several studies have been conducted to determine the autonomic nervous system (ANS) responses to primary tastes using basic taste solutions [12,14,32]. However, no research has been conducted by developing chocolates with different basic tastes to identify the correlations between conscious (self-reported) and unconscious (biometrics) responses when tasting the samples. Chocolate was selected as the sample because it is the most common confectionary worldwide, and there has been a steady increase in consumption in the last decade [33]. Furthermore, sweet and bitter are the two main tastes found in chocolate. However, in this study, the common tastes were modified to determine how it would affect the biometrics of participants and sensory responses. The five basic tastes considered in this study for chocolate and the reactions measured from panelists could help to model specific ANS responses to assess commercial chocolates in the future.

## 2. Materials and Methods

### 2.1. Sample Preparation

Twelve replicates of chocolates (three samples each from salty, sour, sweet, and umami flavored chocolate) were developed in the Sensory laboratory at The University of Melbourne, Australia. All chocolates were poured in a square mold, and the size of each chocolate piece was 1 cm × 1 cm and 7.3 g of weight. From the basic tastes, the use of sucrose to measure the sensitivity to sweetness has been approved by the International Standardization Organization (ISO). Also, sodium chloride (NaCl) for salt taste, monosodium glutamate (MSG) for umami, and citric acid for sour taste have been approved for use in sensory studies [14]. Accordingly, the recommended additives were used in this study for the preparation of the chocolate samples used.

The three concentrations used for different tastes were: 3%, 6%, and 9% for sweet; 2%, 4%, and 6% for salty; 0.5%, 2.5%, and 4% for sour; and 0.5%, 2.5%, and 3.5% for umami. The developed chocolate samples were evaluated in a focus group (*n* = 5) discussion consisting of sensory professionals from The University of Melbourne to select the best concentration of each taste based on intensity. Participants were given two pieces of chocolate from each taste and were asked initially to identify the taste. Once identified, they had to rate the intensity of the specific taste on a 5-point hedonic scale. Finally, they selected the most liked samples out of three concentrations for each taste. The basic taste concentrations selected from each sample are shown in Table 1. These selected samples were prepared by adding the respective weight of additives to 100 g of melted compound milk chocolate (Cadbury, Ringwood, Australia). The chocolate was melted using a microwave with short time increments while stirring in between, until it reached 40–50 °C [34]. Then, the melted chocolate was poured into molds and kept in a refrigerator at 4 °C for 20 min to solidify. Four bars from each chocolate taste were prepared and used for sensory evaluation. Commercially available 70% dark chocolate (Cadbury, Cadbury’s Chocolate Factory, Tasmania, Australia) was used as the bitter chocolate sample.

### 2.2. Sensory Evaluation and Analysis of the Self-Reported (Conscious) Data

Panelists (*n* = 45) between 18 and 55 years old were recruited from a pool of staff and students via email invitations from The University of Melbourne, Australia, who volunteered to participate in the sensory assessment of developed chocolate samples with basic tastes. Panelists received incentives (chocolate and confectionary products) as an appreciation for their participation in the sensory sessions. The experimental procedure was approved by the ethics committee of the Faculty of Veterinary and Agricultural Sciences at The University of Melbourne, Australia (Ethics ID 1545786.2). According to the Power analysis (1 − β > 0.999) performed using Statistical Analysis System (SAS)^®^ Power and Sample Size 14.1 software (SAS Institute Inc., Cary, NC, USA) with the specific data from this study, a sample of 45 participants was sufficient to find significant differences between samples.

Sessions were conducted in individual sensory booths in the sensory laboratory at The University of Melbourne. The temperature of the booths was set between 24 and 25 °C, while the preparation/serving room was set at a temperature of 20 °C. Booths were illuminated with uniform white lighting. The sensory laboratory consists of 20 individual booths, and the panelists did not have access to the kitchen or the serving room. During the sensory session, the samples were served to participants through a hatch with a double sliding door. The booths were equipped with an integrated camera system, which consists of a FLIR^®^ AX8 infrared thermal camera (FLIR^®^ Systems, Wilsonville, OR, USA) coupled with a Bio-Sensory application (App; The University of Melbourne, Melbourne, Vic, Australia) [35] developed for Android tablet PCs (Google; Open Handset Alliance, Mountain View, CA, USA). Panelists were asked to sit 30–45 cm from the tablets during the sessions. The data were gathered from the sensory panel using a 15-cm, non-structured, continuous scale. Chocolate samples (2 pieces from each taste; size: 1 cm × 1 cm and 7.3 g) were served on a tray in a random order with 3-digit random codes for evaluation. Water and crackers were served to panelists to cleanse the palate between each sample.

The questions for self-reported responses and anchored answer options are shown in Table 2. Finally, a check-all-that-apply (CATA) question was given to select all emotions associated with chocolate consumption using an emotion lexicon developed for chocolate [31]. The given emotions are shown in Table 3. Consumers tend to select more positive emotions when compared to negative when explaining their food experiences, which is known as “hedonic asymmetry” [30,36,37]. Hence, the lexicon in Table 3 contains more positive than negative or neutral emotions.

### 2.3. Analysis of the Biometric (Unconscious) Data

Infrared thermal images were used to obtain the ST of participants using a customized code written in Matlab^®^ R2019a (Mathworks Inc., Natick, MA, USA) [35,38]. This algorithm can detect the eye section (area of interest) automatically for each image using the cascade object detector [39]. Videos obtained were used to measure the HR values in beats per minute (BPM) using two Matlab^®^ R2019a codes. The first code magnifies the color using the Eulerian Magnification Algorithm (EMA) developed by the Massachusetts Institute of Technology (MIT, Boston, MA, USA) [40]. From the second code, HR estimations were obtained through skin luminosity changes magnified by EMA. Videos were also used to analyze the FE of participants using the FaceReader™ software (Noldus Information Technology, Wageningen, Netherlands) and average values were obtained as intensities for each emotion. Eight emotions: (i) happy, (ii) sad, (iii) angry, (iv) surprised, (v) scared, (vi) disgusted, (vii) contempt, and (viii) neutral, two emotional dimensions: (i) valence and (ii) arousal, and head orientation in three directions: (i) x, (ii) y, and (iii) z were measured using the FaceReader™ software.

### 2.4. Statistical Analysis

Analysis of Variance (ANOVA) and the post-hoc Tukey simultaneous multiple comparison at 95% confidence intervals were obtained to identify the significant differences (α = 0.05) of the unconscious (biometric) and sensory conscious (self-reported) responses between chocolate with different tastes using Minitab 2017 (Minitab Inc., State College, PA, USA). Multivariate data analysis for all conscious and unconscious data was conducted based on principal component analysis (PCA) using a customized code written in Matlab^®^ R2019a [35]. A correlation matrix was also performed using that same code for all the descriptors used to draw the Principal Component Analysis (PCA) showing the significant (*p* < 0.05) correlations. Analysis of Check-All-That-Apply (CATA) of emotional data was conducted using XLSTAT 2017 (Addinsoft, New York, NY, USA). Cochran’s Q test, correspondence analysis (CA), and principal coordinate analysis (PCoA) were conducted. The Cochran’s Q test can be used to identify if the chocolate samples are associated with any of the emotion-based terms individually.

## 3. Results

### 3.1. Sensory Responses

According to the results from ANOVA for self-reported responses, there were significant differences between the chocolate samples (*p* < 0.05) regarding overall liking (Table 4). All the basic taste intensities (bitterness, saltiness, sourness, sweetness, and umami taste) as well as texture (hardness and smoothness) also showed significant differences (*p* < 0.05) between samples. However, there were no significant differences (*p* ≥ 0.05) between samples for persistence in the mouth. Table 4 shows the results of mean values and standard deviations for the self-reported conscious responses towards the chocolate samples. From Table 4, it can also be observed that overall liking for the sweet sample was significantly higher (*p* < 0.05) than for the salty, sour, and umami samples, but was not significantly different (*p* ≥ 0.05) from the bitter sample. Furthermore, overall liking for the salty (3.71) sample was lower than for the sour (7.11), sweet (8.83), and umami (5.34), being the salty chocolate the least liked. The sweet sample had higher overall liking than the sour, while the umami chocolate had lower overall liking values than the sour and sweet samples. Hence, the sweet sample was the most liked chocolate.

### 3.2. Biometric Responses

Table 5 shows the mean values for the unconscious responses towards the chocolate samples. According to the ANOVA for the ANS responses (FE, ST, and HR) of the sensory session depending on the samples, all FaceReader™ emotions, and dimensions, as well as other biometric results, were significantly different between tasters (*p* < 0.05). However, there were no significant differences (*p* ≥ 0.05) between samples for the FaceReader™ emotions and biometric results. There were significant differences (*p* < 0.05) between samples for head orientation (x, y, and z) results.

### 3.3. Multivariate Analysis and Correlations between Variables

**Figure 1:**
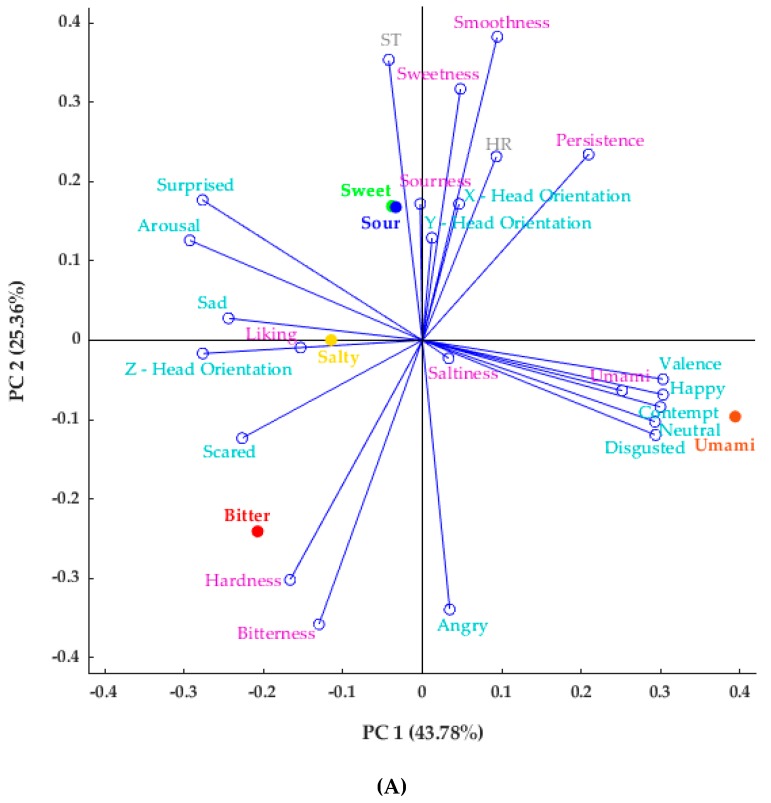

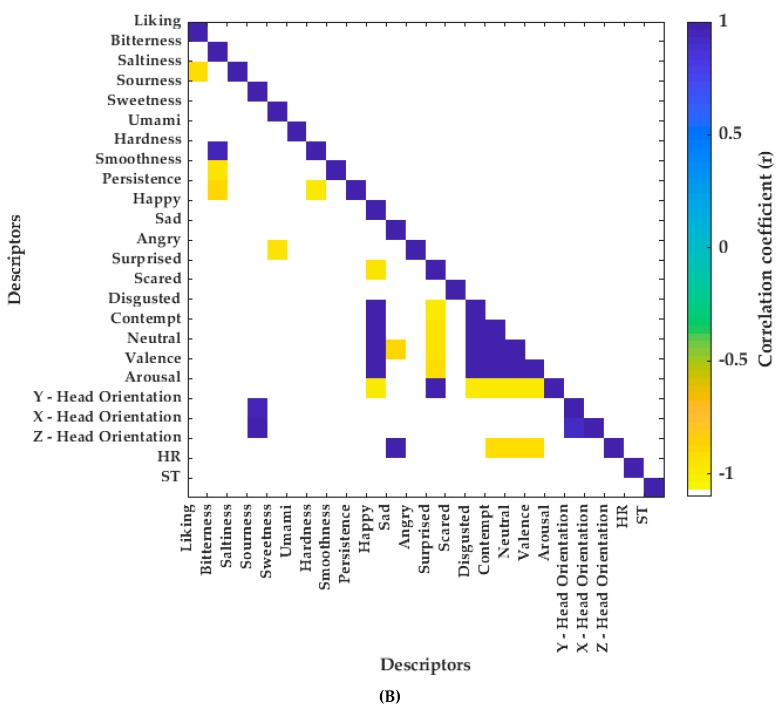
Results of multivariate data analysis. (**A**) Principal Component Analysis (PCA) for FaceReader™, physiological, and sensory parameters of chocolate tasting session, (**B**) Correlation matrix obtained from standardized codes. Only significant correlations (*p* < 0.05) are shown in the figure (HR = heart rate; ST = skin temperature). The color bar shows a minimum value of −1 (yellow) and a maximum value of 1 (blue).

Results of PCA for FaceReader™ (FR), physiological, and sensory parameters are shown in Figure 1A. The principal component 1 (PC 1) explained 43.78% of the data variability, while the principal component 2 (PC 2) explained 25.36%. Hence, the total data variability accounted for 69.14%. Sweet and sour chocolate samples were associated with higher ST and lower FR-angry values. The salty chocolate was positively associated with FR-sad and was negatively related with FR-happy. The umami sample showed higher FR-neutral, FR-happy, FR-valance, FR-contempt, and FR-disgusted. The FR-scared and hardness of the chocolate were related to the bitter chocolate, while it showed lower values for HR, smoothness of chocolate, and persistence of taste on the palate. According to the PCA factor loadings (FL; Table S1), PC 1 is mainly represented by FR-happy (FL = 0.30) and umami (FL = 0.25) on the positive side and arousal (FL = −0.29), and FR-surprised (FL = −0.28) and Z-head orientation (FR = −0.28) on the negative side. On the other hand, PC 2 is mainly represented by smoothness (FL = 0.38) and ST (FL = 0.35) on the positive side and bitterness (FL = −0.36) and FR-angry (FL = −0.34) on the negative side.

Figure 1B shows the correlation matrix for all descriptors found in the PCA. The blue side of the color map shows the positive correlations, while the yellow side depicts the negative correlations, with a significance level of 0.05. In the diagram it can be observed that liking was negatively correlated with saltiness (*r* = −0.91). Sweetness had a negative correlation with FR-angry (*r* = −0.95), while bitterness was negatively correlated to smoothness (*r* = −0.96), and had a positive correlation with hardness (*r* = 0.97), which was also found in the PCA.

### 3.4. Emotional Responses (Self-Reported)

According to the Cochran’s Q test (Table 6), the emotion-based terms “active”, “a little naughty”, “bored”, “bright”, “friendly”, “guilty”, “happy”, “healthy”, “interested”, “natural”, and “pleasurable” were significantly different for each taste, while the other emotions did not show significant differences (*p* ≥ 0.05).

**Figure 2:**
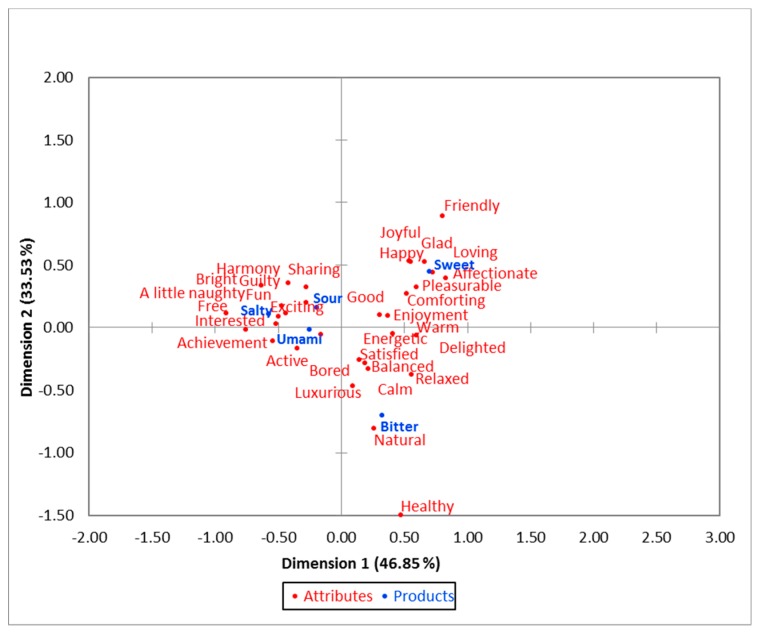
Correspondence analysis from the check-all-that-apply test of the sensory session.

Figure 2 shows the results of the CA from the emotion terms selected when tasting chocolate. Dimension 1 (x-axis) represented 46.85% of the data, while dimension 2 (y-axis) accounted for 33.53% of data variability, with a total of 80.38%. There is a clear separation of the sweet and bitter chocolate samples from other tastes (salty, sour, and umami). The sweet chocolate was associated with terms such as “happy”, “joyful”, “pleasurable”, “affectionate”, “enjoyment”, and “comforting”, while the bitter chocolate was clustered with emotion terms such as “natural”, “luxurious”, “relaxed”, “healthy”, and “satisfied”. The umami chocolate was associated with terms such as “active”, “achievement”, and “bored”, while “interested”, “free”, “excited”, and “a little naughty” were associated with the salty chocolate. The sour chocolate was related to “exciting”, “sharing”, and “guilty”.

**Figure 3:**
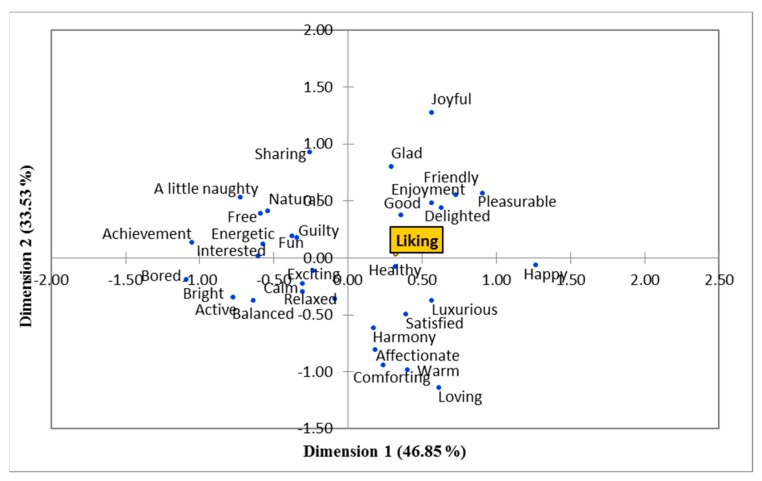
Principal coordinate analysis obtained from the sensory session.

Figure 3 shows the Principal Coordinate Analysis (PCoA) conducted using the overall liking scores and the emotion-based terms of the sensory session. As seen in the figure, liking for chocolate was positively correlated with emotions such as “delighted”, “good”, “enjoyment”, “friendly”, “glad”, “pleasurable”, and “joyful”. On the contrary, “bored”, “bright”, “active”, “balanced”, “relaxed”, “calm”, and “exciting” were negatively correlated to liking.

## 4. Discussion

### 4.1. Self-Reported Sensory Responses

Humans have an innate liking for sweetness [41,42,43], which is also shown in the results of the present study because the sweet chocolate sample was rated higher in liking by participants. Least overall liking for salty chocolate may be due to the health concerns, such as hypertension, as well as strokes and cardiovascular diseases that can be developed by hypertension usually caused by high salt intake [44]. The bitter chocolate (dark) had the second highest overall liking after the sweet sample, which may be owing to the participants perceptions of the potential health benefits due to the antioxidants available in dark chocolate. This was also seen from the present study because as per the correspondence analysis, dark chocolate was associated with terms such as “healthy” and “natural” in CATA. Previous research has shown that there is a correlation between dark chocolate consumption and reduction of cardiovascular diseases by acting against low-density lipoprotein oxidation and free radicals [45,46,47,48,49]. Consumers also responded to compounds that give bitterness differently. For example, phenylthiocarbamide and 6-n-propylthiouracil taste bitter to some and tasteless to others [50]. This may be a reason why people prefer bitterness in some foods and not in other foods and beverages.

The intensity of bitterness, saltiness, sourness, sweetness, and umami taste was highest for their respective chocolate taste. This indicated that the panelists detected the respective basic taste correctly. The texture of the chocolate is affected due to the variations in the particle size distribution [51]. In this study, the hardness and smoothness showed significant differences between chocolate samples, this may be due to the differences in the particle size of each sample, which contain different additives. The use of milk and dark chocolate having different ingredients also can be a reason for the variations in texture between samples. Cocoa butter gives a smooth texture to chocolate, which also has a rich mouthfeel due to the increased fat content. This also reduces the bitterness of chocolate. The addition of milk powder also reduces the hardness of chocolate [52]. This is in accordance with the results of this study because bitter chocolate showed positive correlations to hardness and was negatively correlated to smoothness. Ingredient composition also affects the texture of chocolate, which complied with the results of this study because it showed significantly different mean values for hardness and smoothness of the chocolate samples with different basic tastes, which were produced with different additives [53].

### 4.2. Biometric Responses

Researchers are currently interested in using both implicit and explicit measures to assess sensory responses to food due to limitations in traditional methods. The questionnaire-based conventional methods rely on cognitive analysis, which is unable to predict the spontaneous emotional responses [32]. Additionally, the implicit measures are shown to be valuable and can be related to long-term customer acceptance of food [54]. According to a review conducted using 134 experimental studies, the ANS responses are stated as a critical component in emotional responses [28]. As shown by Rousmans [32], ANS responses to four primary tastes (sweet, salty, sour, and bitter) may be used as a reference for the hedonic analysis of food tasting. Similar to the current study, several studies have been conducted using self-reported responses and biometrics to determine the sensory responses of food products more accurately [7,9,12,27,38,55].

Among the most common ANS responses used to assess consumer reactions to food products are HR and ST [27,56]. The HR responses are a combination of parasympathetic (safe conditions) and sympathetic (stress conditions) activities [57]. Gonzalez Viejo et al. [13] found a decrease in HR when consumers tasted beer samples with higher bitterness, while Rousmans et al. [32] reported that ST was higher when tasting citric acid. In the present study, a similar trend was found, as consumer HR was lower with bitter taste chocolates and ST increased when tasting the sour chocolate (Figure 1A). Some studies have reported that there are weak ANS responses to sweet taste compared to the other basic tastes, mainly due to the habituation of the organism to sweetness [32,58]. However, other studies have shown that the assessment of both ANS responses and FE are able to provide more information about the unconscious responses from consumers when tasting different food products [7,9,13,27]. Moreover, it is shown that conscious responses alone may be insufficient to identify the aspects that affect liking, hence a combination of implicit and explicit measures, as conducted in this study, may contribute to a better understanding of liking and complete eating behavior [59].

None of the biometric responses showed significant differences between the chocolate samples with different tastes, which is in accordance with previous studies conducted using chocolate [7] and beer [38]. Due to the absence of social interactions, there were no detectable differences in FE. This study was conducted in individual sensory booths, and according to previous research, these isolated conditions did not show significant variations in facial reactions [7]. Hence, the trends of the mean values of the “neutral” emotion showed the highest intensity for all samples. Another study conducted using smoked ham showed similar results to this study with high values for “neutral” emotion [60]. Another study was conducted using implicit and explicit measures to identify the relationship of food perception in different test locations—under laboratory conditions, and at home. It was shown that there were no significant variations in liking scores between test location but there were between samples. In contrast, there were systematic effects on implicit measures based on test location, sample, and repeated consumption, and showed intense FE at home [54]. This further explains the expression of emotions at different tasting locations. However, the present study showed contradicting results to a study conducted recently using FaceReader™ and hedonic measurements, where higher values were obtained for “happy” emotion in chocolate and “neutral” in bakery products [61].

According to previous studies, the ST was negatively correlated with FR-angry [7]. These results were also seen from the current study in the PCA (Figure 1A). The ST showed a positive association with the sweet chocolate sample, which was the most liked product, and these results are in accordance with the results shown in previous studies, where the temperature was higher for liked food than for disliked food [27]. Although salty chocolate, the least liked sample, was associated with FR-sad, a negative emotion, the highly liked samples were not showing associations to the positive emotions. Hence, this was in accordance with previous studies, which showed that although FE correctly interprets negative food preferences, they do not perform as well for positive food likings [27,62]. It is important to measure emotional responses in addition to self-reported responses to obtain a better representation of acceptability to food [63,64,65].

### 4.3. Emotional Responses (Self-Reported)

According to an emotion lexicon developed for milk chocolate (sweet) in a previous study by Gunaratne et al. [31], the highest frequencies of responses to chocolates were obtained by emotions such as “happy”, “joyful”, “pleasurable”, “enjoyment”, and “comforting”. Similarly, as seen in the CA, those emotion-based terms were selected for the sweet sample in this study as well. Also, there were similarities in the terms selected for the bitter sample in this study (”natural”, “relaxed”, “healthy”, “satisfied”, and “luxurious”) and terms with highest frequency of selection in the lexicon developed for dark chocolate in the study conducted by Gunaratne et al. [31]. Furthermore, the separation of these terms for the sweet and bitter chocolate samples in this study were also in accordance with another study conducted using milk and dark chocolate [63]. As per the PCoA, liking was positively correlated with the terms “delighted”, “good”, “enjoyment”, “friendly”, “glad”, “pleasurable”, and “joyful”, which were all considered as positive in emotion-based studies conducted previously [30,66].

The environment in which tasting takes place influences the emotions of the tasters [67]. For example, in a wine study, Danner et al. [68] reported that positive emotions were significantly higher in a restaurant environment when compared to laboratory conditions. Similar results were obtained in a sensory study conducted using yogurt [69]. Moreover, positive emotions significantly increased and negative emotions were decreased in a natural environment when compared to controlled conditions when tasting beer [70]. Hence, the controlled conditions in this study might have been a reason for getting a negative correlation between some positive emotions and liking. Furthermore, according to the CA, the least liked sample salty was associated with emotions such as “a little naughty” and “guilty”, which were identified as negative and unclassified emotions in previous studies [31].

Industries are currently shifting towards the development of different types of food, for example incorporating aroma and flavor for beverages through encapsulation [71], enrichment of probiotic bacteria to dark chocolate [72], development of vanilla flavor encapsulated microcapsules that can be used for food [73], and fortification of dark chocolate with microencapsulated phytosterols [74]. The proposed methodology in this study can aid food industries in the development of new products incorporating novel flavors to chocolate, such as chili, sea salt, and bacon, among others. It is important to make sure that these novel food products will be successful in the market, and sensory evaluation plays a major role in this aspect. Hence, the implicit and explicit measures used in this study may be incorporated to evaluate the acceptability of new food products.

## 5. Conclusions

The consumers overall liking was highest for the sweet chocolate, which was determined by the conscious self-reported responses as well as the unconscious biometric and emotional responses. Conventional sensory tests rely on using questionnaires and poorly predict consumer acceptance for new products, which contribute to the high market failure rate. Implicit autonomic nervous system responses may provide a better interpretation of preferences for specific food properties than many explicit measurements. Analysis of facial expressions along with sensory evaluation might help to understand the influence of food properties in eliciting emotions, which lead to determine consumer preferences. Hence, the use of self-reported responses HR, ST, and FE, and emotions in combination may be used as a novel sensory technique to assess the acceptability of consumers to chocolate. Furthermore, the methods used in this study can be used to obtain quantitative measurements of complicated sensory responses to food. This may also be applied to determine the acceptability of the incorporation of microcapsules for food products and different flavors to chocolate, such as chili or other non-conventional ones, which food industries would be keen to introduce. Moreover, similar studies may be conducted in different test locations to determine the relationship of FE when tasting food products under social interactions and laboratory conditions. Further studies may be conducted to develop machine learning models using the sensory and physiological data as a rapid screening technique in sensory analysis to determine the best chocolate samples in new product development.

## Figures and Tables

**Table 1:** Concentration of additives used for the production of selected chocolate samples.

Basic Taste	Additive	Concentration (%)
Salty	Salt (SAXA, Cheetham Salt Limited, Melbourne, Australia)	4.0
Sour	Citric acid (McKenzie’s, Ward Mckenzie Pty Ltd., Altona, Australia)	0.5
Sweet	Sucrose (CSR, CSR Limited, North Ryde BC, NSW, Australia)	6.0
Umami	Monosodium glutamate (Spice Supreme, Spice Supreme®, Bayonne, NJ, USA)	3.5

**Table 2:** Questions for self-reported responses and given answer options.

Question	Anchors in Continuous Line Scale
What flavor do you perceive?	Cocoa/vanilla/caramel/sweet/buttery/milky/burnt
What taste do you perceive?	Bitter/salty/sour/sweet/umami
Rate the intensity of detected taste	Absent/low/medium/high
Rate the hardness at first bite	Soft/medium/hard
Rate the smoothness of the sample	Low/medium/high
Rate the overall liking of the sample	0—Dislike extremely/7.5—Neither like nor dislike/15—Like extremely
Indicate the persistence of taste in mouth (in seconds)	0/2/4/>6
Willingness to taste another piece of chocolate	Not at all/Maybe/Yes, definitely

**Table 3:** Lexicon of emotions for chocolate given for the Check-All-That-Apply (CATA) question.

Achievement	Active	Affectionate	A little naughty
Balanced	Bored	Bright	Calm
Comforting	Delighted	Energetic	Enjoyment
Exciting	Free	Friendly	Fun
Glad	Good	Guilty	Happy
Harmony	Healthy	Interested	Joyful
Loving	Luxurious	Natural	Pleasurable
Relaxed	Satisfied	Sharing	Warm

**Table 4:** Mean values for conscious responses (basic taste intensity, texture, and other self-reported responses) of chocolate samples.

Sample	Self-Reported Responses		Basic Taste Intensity					Texture	
	Liking	Persistence	Bitterness	Saltiness	Sourness	Sweetness	Umami Taste	Hardness	Smoothness
Bitter	8.7 ± 3.69 ^a^	11.07 ± 3.97 ^a^	10.16 ± 3.62 ^a^	2.25 ± 2.91 ^c,d^	2.35 ± 3.40 ^b,c^	4.31 ± 3.34 ^c^	3.28 ± 4.13 ^c^	12.87 ± 2.16 ^a^	4.96 ± 3.86 ^c^
Salty	3.71 ± 4.13 ^c^	11.51 ± 3.58 ^a^	3.15 ± 3.90 ^b^	13.37 ± 2.25 ^a^	4.17 ± 4.39 ^b^	5.12 ± 3.69 ^c^	6.4 ± 4.70 ^a,b^	6.1 ± 2.95 ^b^	7.47 ± 3.21 ^b^
Sour	7.11 ± 3.23 ^a,b^	11.46 ± 3.51 ^a^	2.17 ± 2.85 ^b,c^	3.93 ± 3.82 ^c^	9.54 ± 4.29 ^a^	9 ± 3.61 ^b^	4.79 ± 3.77 ^b,c^	5.6 ± 2.93 ^b^	9.73 ± 2.71 ^a^
Sweet	8.83 ± 3.40 ^a^	11.48 ± 2.88 ^a^	0.99 ± 2.35 ^c^	1.84 ± 2.48 ^d^	1.15 ± 2.05 ^c^	11.95 ± 3.39 ^a^	2.56 ± 3.22 ^c^	6.04 ± 3.13 ^b^	9.09 ± 3.13 ^a,b^
Umami	5.34 ± 3.71 ^b,c^	11.61 ± 3.54 ^a^	2.82 ± 4.07 ^b,c^	7.02 ± 3.17 ^b^	3.31 ± 3.67 ^b,c^	7.85 ± 4.45 ^b^	7.43 ± 5.25 ^a^	5.71 ± 2.96 ^b^	7.71 ± 2.93 ^b^

^a–d^ Means with different letters for each parameter indicate significant differences (P<0.05) from the Tukey’s Studentized Range (HSD) test.

**Table 5:** Mean values for unconscious responses of chocolate samples.

**Sample**	**FaceReader™ emotions**							
	**Happy ^NS^**	**Sad ^NS^**	**Angry ^NS^**	**Surprised ^NS^**	**Scared ^NS^**	**Disgusted ^NS^**	**Contempt ^NS^**	**Neutral ^NS^**
Bitter	0.03 ± 0.04	0.10 ± 0.09	0.27 ± 0.23	0.18 ± 0.18	0.05 ± 0.10	0.02 ± 0.04	0.07 ± 0.05	0.30 ± 0.10
Salty	0.04 ± 0.09	0.14 ± 0.17	0.28 ± 0.23	0.19 ± 0.19	0.05 ± 0.10	0.04 ± 0.04	0.06 ± 0.05	0.26 ± 0.10
Sour	0.04 ± 0.08	0.12 ± 0.14	0.26 ± 0.22	0.18 ± 0.19	0.05 ± 0.09	0.02 ± 0.09	0.08 ± 0.07	0.30 ± 0.14
Sweet	0.05 ± 0.10	0.12 ± 0.14	0.26 ± 0.24	0.20 ± 0.20	0.03 ± 0.06	0.03 ± 0.06	0.08 ± 0.06	0.29 ± 0.12
Umami	0.03 ± 0.06	0.11 ± 0.12	0.29 ± 0.25	0.17 ± 0.14	0.06 ± 0.09	0.28 ± 0.04	0.06 ± 0.04	0.28 ± 0.10
**Sample**	**FaceReader™ dimensions**		**Biometrics**		**Head orientations**			
	**Valence ^NS^**	**Arousal ^NS^**	**Heart rate (BPM) ^NS^**	**Skin temperature (°C) ^NS^**	**X–Head orientation**	**Y–Head orientation**	**Z–Head orientation**	
Bitter	−0.35 ± 0.19	0.34 ± 0.12	69.19 ± 18.36	34.97 ± 36.65	−1.56 ± 0.31 ^d^	6.49 ± 0.71 ^b^	0.38 ± 0.31 ^b^	
Salty	−0.38 ± 0.24	0.32 ± 0.10	70.94 ± 6.65	35.83 ± 37.30	−0.39 ± 0.26 ^c^	2.03 ± 0.77 ^c^	1.45 ± 0.32 ^a^	
Sour	−0.34 ± 0.24	0.34 ± 0.10	69.95 ± 10.48	35.70 ± 36.90	3.19 ± 0.48 ^a^	22.64 ± 0.59 ^a^	−1.10 ± 0.45 ^c^	
Sweet	−0.33 ± 0.24	0.45 ± 0.12	73.14 ± 11.03	35.77 ± 37.32	−1.66 ± 0.21 ^d^	2.70 ± 0.22 ^c^	−0.56 ± 0.16 ^c^	
Umami	−0.37 ± 0.21	0.46 ± 0.10	71.17 ± 9.02	35.24 ± 1.72	0.19 ± 0.09 ^b^	7.64 ± 0.26 ^b^	−3.54 ± 0.21 ^d^	

^a–d^ Means with different letters for each parameter indicate significant differences (*p* < 0.05) by the Tukey’s Studentized Range (HSD) test. NS = no significant difference (*p* ≥ 0.05) from the Tukey’s Studentized Range (HSD) test.

**Table 6:** Cochran’s Q test results for each emotion-based term of chocolate with basic tastes.

Attributes	p-values	Bitter	Salty	Sour	Sweet	Umami
Achievement	0.05	0.02 ^a^	0.12 ^a^	0.02 ^a^	0.00 ^a^	0.10 ^a^
**Active**	**0.01**	**0.10 ^a^**	**0.19 ^a^**	**0.17 ^a^**	**0.00 ^a^**	**0.05 ^a^**
Affectionate	0.09	0.02 ^a^	0.00 ^a^	0.00 ^a^	0.10 ^a^	0.05 ^a^
**A little naughty**	**0.02**	**0.05 ^a^**	**0.24 ^a^**	**0.21 ^a^**	**0.07 ^a^**	**0.26 ^a^**
Balanced	0.48	0.14 ^a^	010 ^a^	0.02 ^a^	0.10 ^a^	0.10 ^a^
**Bored**	**0.02**	**0.24 ^a^**	**0.33 ^a^**	**0.19 ^a^**	**0.07 ^a^**	**0.31 ^a^**
**Bright**	**0.02**	**0.00 ^a^**	**0.14 ^a^**	**0.17 ^a^**	**0.02 ^a^**	**0.07 ^a^**
Calm	0.20	0.17 ^a^	0.05 ^a^	0.05 ^a^	0.10 ^a^	0.14 ^a^
Comforting	0.13	0.07 ^a^	0.02 ^a^	0.10 ^a^	0.17 ^a^	0.05 ^a^
Delighted	0.37	0.14 ^a^	0.05 ^a^	0.07 ^a^	0.17 ^a^	0.10 ^a^
Energetic	0.73	0.10 ^a^	0.14 ^a^	0.07 ^a^	0.07 ^a^	0.07 ^a^
Enjoyment	0.49	0.12 ^a^	0.10 ^a^	0.07 ^a^	0.19 ^a^	0.10 ^a^
Exciting	0.13	0.05 ^a^	0.14 ^a^	0.17 ^a^	0.02 ^a^	0.14 ^a^
Free	0.20	0.00 ^a^	0.05 ^a^	0.00 ^a^	0.00 ^a^	0.05 ^a^
**Friendly**	**0.01**	**0.00 ^a^**	**0.00 ^a^**	**0.07 ^a^**	**0.12 ^a^**	**0.00 ^a^**
Fun	0.34	0.05 ^a^	0.17 ^a^	0.10 ^a^	0.05 ^a^	0.10 ^a^
Glad	0.05	0.02 ^a^	0.00 ^a^	0.10 ^a^	0.14 ^a^	0.05 ^a^
Good	0.14	0.12 ^a^	0.02 ^a^	0.17 ^a^	0.17 ^a^	0.07 ^a^
**Guilty**	**0.01**	**0.12 ^a^**	**0.43 ^a^**	**0.19 ^a,b^**	**0.21 ^a^**	**0.19 ^a,b^**
**Happy**	**0.001**	**0.05 ^a^**	**0.10 ^a^**	**0.05 ^a^**	**0.29 ^a^**	**0.07 ^a^**
Harmony	0.12	0.00 ^a^	0.05 ^a^	0.05 ^a^	0.05 ^a^	0.12 ^a^
**Healthy**	**0.000**	**0.26 ^b^**	**0.02 ^a,b^**	**0.02 ^a,b^**	**0.00 ^a^**	**0.00 ^a^**
**Interested**	**0.001**	**0.12 ^a,b^**	**0.29 ^a,b^**	**0.36 ^b^**	**0.02 ^a^**	**0.24 ^a,b^**
Joyful	0.09	0.02 ^a^	0.00 ^a^	0.07 ^a^	0.12 ^a^	0.02 ^a^
Loving	0.16	0.02 ^a^	0.02 ^a^	0.00 ^a^	0.10 ^a^	0.02 ^a^
Luxurious	0.16	0.19 ^a^	0.05 ^a^	0.12 ^a^	0.05 ^a^	0.10 ^a^
**Natural**	**0.000**	**0.31 ^b^**	**0.02 ^a^**	**0.07 ^a,b^**	**0.05 ^a,b^**	**0.14 ^a,b^**
**Pleasurable**	**0.01**	**0.10 ^a^**	**0.05 ^a^**	**0.12 ^a^**	**0.24 ^a^**	**0.02 ^a^**
Relaxed	0.10	0.14 ^a^	0.02 ^a^	0.05 ^a^	0.10 ^a^	0.02 ^a^
Satisfied	0.20	0.17 ^a^	0.02 ^a^	0.10 ^a^	0.10 ^a^	0.17 ^a^
Sharing	0.68	0.00 ^a^	0.05 ^a^	0.05 ^a^	0.02 ^a^	0.05 ^a^
Warm	0.28	0.10 ^a^	0.02 ^a^	0.02 ^a^	0.12 ^a^	0.05 ^a^

Terms in bold are significantly different for each chocolate sample. Two terms sharing the same superscript letter do not differ significantly, while the terms with no common superscript letter differ significantly within a column.

## Supplementary Materials

The following are available online at https://www.mdpi.com/2304-8158/8/7/243/s1. Table S1: Factor loadings from the principal component analysis for descriptors used in Figure 1. The first two principal components (PC 1 and PC 2) are shown.
