# Supplementary material for: Physiological Responses to Basic Tastes for Sensory Evaluation of Chocolate Using Biometric Techniques
Source: Foods. 2019 Jul 5;8(7):243. doi: 10.3390/foods8070243 (PMC6679144; doi:10.3390/foods8070243)
Supplement: Supplementary file 1 [file foods-08-00243-s001.pdf]

Supplementary Materials:

Table S1. Factor loadings from the principal component analysis for descriptors used in Figure 1. The first two principal components (PC 1 and PC 2) are shown.

Attribute	PC 1	PC 2
Liking	−0.15	−0.01
Bitterness	−0.13	−0.36
Saltiness	0.03	−0.02
Sourness	−0.003	0.17
Sweetness	0.05	0.32
Umami	0.25	−0.06
Hardness	−0.17	−0.30
Smoothness	0.09	0.38
Persistence	0.21	0.23
Happy	0.30	−0.07
Sad	−0.24	0.03
Angry	0.03	−0.34
Surprised	−0.28	0.18
Scared	−0.23	−0.12
Disgusted	0.29	−0.12
Contempt	0.30	−0.08
Neutral	0.29	−0.10
Valence	0.30	−0.05
Arousal	−0.29	0.13
Y—Head Orientation	0.01	0.13
X—Head Orientation	0.05	0.17
Z—Head Orientation	−0.28	−0.02
HR	0.09	0.23
ST	−0.04	0.35
